# Complete resolution of stage II avascular necrosis affecting three joints by hyperbaric oxygen in a patient with sickle cell disease: A case report

**DOI:** 10.3389/fmed.2022.1063255

**Published:** 2022-11-30

**Authors:** Awni Alshurafa, Mohammad Elhissi, Mohamed A. Yassin

**Affiliations:** ^1^Department of Hematology, Hamad Medical Corporation, Doha, Qatar; ^2^Clinical Imaging Department, Hamad Medical Corporation, Doha, Qatar

**Keywords:** sickle cell disease, avascular necrosis, hyperbaric oxygen, osteonecrosis, vaso-occlusive crisis

## Abstract

Avascular necrosis (AVN) or joint osteonecrosis is a debilitating complication of sickle cell disease, increasing the disease burden on both patients and healthcare systems. AVN can be radiologically categorized into early and late stages depending on the extent of the disease. Management of AVN is challenging and controversial. Generally, it includes conservative measures for early disease to preserve the joint for as long as possible and surgical management for more advanced diseases. Hyperbaric oxygen (HBO) therapy can be used as primary or adjunctive therapy for different medical disorders. Currently, the main rule of HBO in AVN is an adjunctive therapy to control symptoms and improve the quality of life of a patient; however, the concept of using HBO as a primary treatment choice for AVN in patients with sickle cell disease is not well evaluated yet. In this case study, we reported a 15-year-old boy with sickle cell disease who was suffering from stage II AVN in bilateral femoral and right shoulder joints. A total of 57 sessions of HBO resulted in the complete resolution of AVN in post-treatment MRI.

##  Introduction

Sickle cell disease is an inherited disorder of the globulin chains and the most common genetic disorder in the United States (1). Patients with sickle cell disease (SCD) are at a high risk of a variety of serious organ system complications. Recurrent vaso-occlusive crisis, chronic hemolysis, hypercoagulability, and fat emboli are the main mechanisms of end-organ injury in SCD (2–5). Avascular necrosis (AVN) is a well-reported complication that affects around 10% of patients with SCD (6). It can result in severe pain and loss of joint function affecting the patient’s quality of life. AVN can be radiologically categorized into early and late stages depending on the extent of the disease (7).

The management of AVN is challenging and controversial. Generally, it includes conservative measures for early disease to preserve the joint for as long as possible, while surgical options are usually kept for more advanced diseases (8). Conservative measures may include physical therapy, offloading as tolerated, and pain management. Low-molecular-weight heparin may decrease the progression rate of idiopathic osteonecrosis from the pre-collapse to the collapsed stage (9, 10).

Hyperbaric oxygen (HBO) is a treatment modality in which individuals breathe 100 % of oxygen which may improve AVN by increasing the oxygen supply to joints. Currently, its main role in AVN is to control joint pain, improve the range of motion, and delay joint loss (11, 12). Some studies reported radiographic improvement in AVN stages I and II with HBO (13). However, the complete resolution of stage II AVN by HBO has not been reported before.

In this case study, we reported a 15-year-old boy with SCD, who was suffering from stage II avascular necrosis in both hips and right shoulder joints. A total of Fifty-seven sessions of HBO resulted in the complete resolution of AVN in post-treatment MRI.

## Case report

**FIGURE 1:**
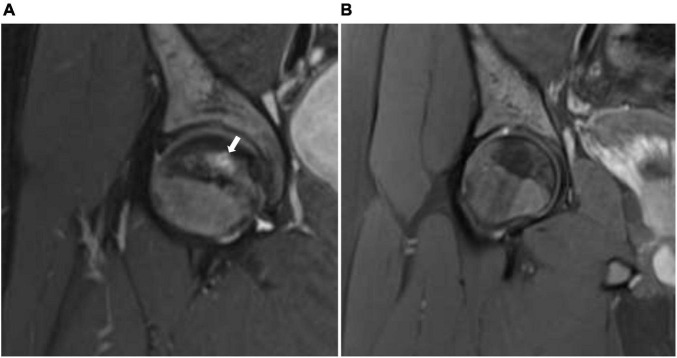
**(A)** Coronal STIR (short T1 inversion recovery) of the right hip before treatment with HBO showing geographic subarticular area of high-signal intensity (arrow) with no collapse of the head of the femur (stage II). **(B)** Coronal STIR (short T1 inversion recovery) of the right hip after treatment showing complete resolution of the previously seen subarticular high-signal intensity suggests complete radiological resolution of avascular necrosis.

**FIGURE 2:**
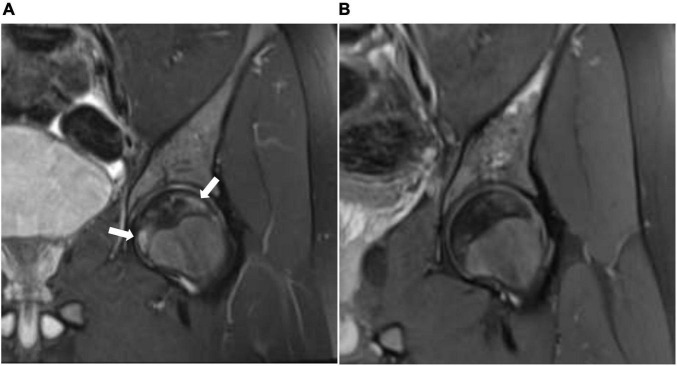
**(A)** Coronal STIR (short T1 inversion recovery) of the left hip before treatment with HBO showing geographic subarticular area of high-signal intensity (arrows) with no collapse of the head of the femur (stage II). **(B)** Coronal STIR (short T1 inversion recovery) of the left hip after treatment showing complete resolution of the previously seen subarticular high-signal intensity suggests complete radiological resolution of avascular necrosis.

**FIGURE 3:**
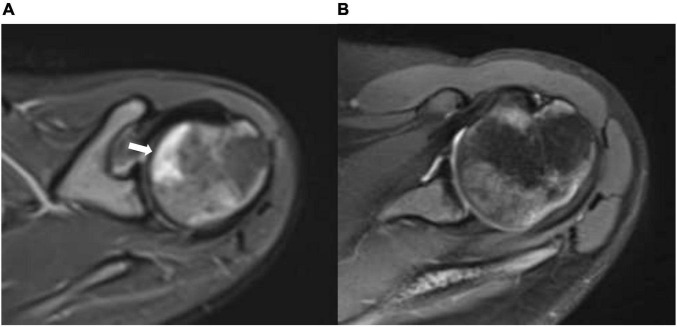
**(A)** Axial STIR (short T1 inversion recovery) of the right shoulder before treatment with HBO showing geographic subarticular area of high-signal intensity (arrow) with no collapse of the head of the femur (stage II). **(B)** Axial STIR (short T1 inversion recovery) of the right shoulder after treatment showing complete resolution of the previously seen subarticular high-signal intensity suggests complete radiological resolution of avascular necrosis.

We report a 15-year-old boy who was suffering from a known case of non-transfusion-dependent SCD (hemoglobin SS) and was on hydroxyurea of 1,000 mg daily. His disease course was complicated by a chronic aching pain and a limited range of motion in both hip and right shoulder joints. An MRI showed geographic subarticular high-signal intensities in STIR sequences with no femoral or humeral head collapse of both hip joints and right shoulder joint in keeping with a stage II AVN of both femurs and right shoulder (Figures 1A, 2A, 3A). Apart from SCD, there were no other risk factors for AVN.

Conservative measures were tried with pain management, offloading, and physiotherapy without significant clinical improvement. Thus, the patient and family were given counseling about HBO to control the joint pain and reduce analgesic use and they agreed to the treatment.

A total of 57 sessions of HBO therapy was given; it was five sessions per week that lasted for 2 h with a pressure of 2.5 pounds per square inch. The treatment was well-tolerated, and the course was uneventful of any side effects. The number of sessions was guided by clinical improvement.

The patient showed significant clinical improvement in the form of pain resolution and range of motion improvement. Post-treatment MRI showed total resolution of the previously noted geographic subarticular high-signal intensities of both hip joints and right shoulder joint denoting complete resolution (Figures 1B, 2B, 3B) of the AVN in affected joints.

## Discussion

Avascular necrosis or osteonecrosis is a debilitating complication of SCD, increasing the disease burden on both patients and healthcare systems. Radiologically, AVN can be divided into pre-collapse and post-collapse of the subchondral surface. Although the management of AVN in patients with SCD is controversial, initially, conservative measures are usually tried because it is less invasive and may be effective for some individuals. Patients and their families are increasingly involved in making treatment decisions (14, 15).

Hyperbaric oxygen therapy is a treatment modality that can be used as primary or adjunctive therapy for different medical disorders. Although the therapy is not approved worldwide, the main rule of HBO in AVN is an adjunctive treatment to control symptoms and to improve the quality of life of a patient; however, the concept of using HBO as a primary treatment choice for AVN in patients with SCD is not well established yet (13, 16).

In a recently published systematic review and meta-analysis assessing the use of HBO in femoral head AVN, regardless of the underlying etiology, Paderno et al. showed that patients with femoral head AVN managed with HBO can achieve a significant clinical improvement in the form of pain reduction and improvement in the range of hip motion; however, the radiological outcome was not described (13). In a double-blind, randomized, controlled, prospective study evaluating HBO therapy in 20 patients with femoral head necrosis, Camporesi et al. showed that besides clinical improvement, there was a continuous radiological improvement in most studied patients at 7 years of follow-up; however, most of the radiographic changes were seen between the baseline MRI and the 12-month post-treatment follow-up images (17). In an SCD-related AVN, Shier et al. reported three cases of pre-collapse femoral head AVN. Of the three cases, two cases showed significant clinical and radiological improvement with HBO (11).

The exact mechanism of the therapeutic effects of HBO in AVN is not yet fully understood. HBO acts by giving oxygen at high atmospheres of pressure, resulting in an increased level of dissolved oxygen in the plasma, and in turn, more oxygen reaches the tissues (18). At the molecular level, the main players in bone turnover are specific cytokines osteoprotegerin (OPG), receptor activator of NF-kB (RANK), and its ligand (RANKL). Any change in the OPG/RANKL/RANK interaction will lead to a shift either to bone formation or resorption. Previous studies showed that there were interactions between inflammatory factors such as interleukin-1 beta (IL-1b), interleukin-6 (IL-6), tumor necrosis factor-alpha (TNF-a), and the OPG/RANK/RANKL. Bosco et al. showed a significant decrease in TNF-a and IL-6 plasma levels with HBO therapy over time. This decrease in inflammatory markers corresponded to reductions in joint pain and bone marrow edema on imaging (19–21).

Hyperbaric oxygen therapy is generally well-tolerated, and most side effects are mild and reversible. Pressure is usually maintained between 2.5 and 3.0 atm, while session number and duration are usually decided according to the indication; one or two sessions may be needed in acute settings, while more extended treatment is usually required for chronic medical conditions (22). The role of novel therapies in the sickle cell-related AVN is still not clear (23–25). In our case, the patient was complaining of chronic aching pain in three joints and requiring frequent analgesic use. HBO was started to control pain and delay joint loss. However, clinical and radiological follow-up showed a complete resolution of previously reported stage II AVN.

In conclusion, HBO therapy may be used as a primary treatment modality in an SCD-related AVN in both controlling symptoms and ameliorating radiological findings; however, further studies are needed to confirm these findings.

## Data availability statement

The original contributions presented in this study are included in the article/supplementary material, further inquiries can be directed to the corresponding author.

## Ethics statement

Written informed consent was obtained from the individual for the publication of any potentially identifiable images or data included in this article.

## Author contributions

All authors listed have made a substantial, direct, and intellectual contribution to the work, and approved it for publication.
